# Mir-135a enhances cellular proliferation through post-transcriptionally regulating PHLPP2 and FOXO1 in human bladder cancer

**DOI:** 10.1186/s12967-015-0438-8

**Published:** 2015-03-13

**Authors:** Xiao Peng Mao, Luo Sheng Zhang, Bin Huang, Shi Ying Zhou, Jun Liao, Ling Wu Chen, Shao Peng Qiu, Jun Xing Chen

**Affiliations:** Department of Urology, the First Affiliated Hospital, Sun Yat-Sen University, Guangzhou, 510080 PR China; Oncology Department, PLA458 Hospital, Guangzhou, 510000 China

**Keywords:** Bladder cancer, miR-135a, PHLPP2, FOXO1, Proliferation

## Abstract

**Background:**

Bladder cancer is the most common malignancy in urinary system and the ninth most common malignancy in the world. MicroRNAs (miRNAs) are small, non-coding RNAs that regulate gene expression by targeted repression of transcription and translation and play essential roles during cancer development. We investigated the expression of miR-135a in bladder cancer and explored its bio-function during bladder cancer progression.

**Methods:**

The expression of miR-135a in bladder cancer cells and tissues are performed by using Real-time PCR assay. Cell viability assay (MTT assay), colony formation assay, anchorage-independent growth ability assay and Bromodeoxyuridine labeling and immunofluorescence (BrdUrd) assay are used to examine cell proliferative capacity and tumorigenicity. Flow cytometry analysis is used to determine cell cycle progression. The expressions of p21, p27, CyclinD1, Ki67, PHLPP2 and FOXO1 are measured by Western blotting assay. Luciferase assay is used to confirm whether FOXO1 is the direct target of miR-135a.

**Results:**

miR-135a is upregulated in bladder cancer cells and tissues. Enforced expression of miR-135a promotes bladder cancer cells proliferation, whereas inhibition of miR-135a reverses the function. Furthermore, for the first time we demonstrated PHLPP2 and FOXO1 are direct targets of miR-135a and transcriptionally down-regulated by miR-135a. Suppression of PHLPP2 or FOXO1 by miR-135a, consisted with dysregulation of p21, p27, Cyclin D1 and Ki67, play important roles in bladder cancer progression.

**Conclusion:**

Our study demonstrates that miR-135a promotes cell proliferation in bladder cancer by targeting PHLPP2 and FOXO1, and is performed as an onco-miR.

**Electronic supplementary material:**

The online version of this article (doi:10.1186/s12967-015-0438-8) contains supplementary material, which is available to authorized users.

## Introduction

Bladder cancer is the most common malignancy involving the urinary system and more than 350,000 new cases of bladder cancer are diagnosed globally each year [1,2]. Bladder cancer is the fourth most common cancer in males and ninth most common in females; it is the ninth most common malignancy overall [2,3]. In middle-aged and elderly men, bladder cancer is the second most prevalent malignancy after prostate cancer [4]. Due to the high occurrence and mortality, accurate and effective diagnostic and therapeutic methods for bladder cancer, based on a comprehensive understanding of bladder cancer progression, are urgently required. Increasing attention is being paid to the biological behavior and mechanisms of bladder cancer development.

The PHLPP (Pleckstrin Homology (PH) domain leucine-rich repeat protein phosphatase) isoforms comprises three members, PHLPP1α, PHLPP1β, and PHLPP2, which are essential regulators of Akt serine-threonine kinases [5-7]. PHLPP2, as one member of PHLPP, attenuate the amplitude of Akt signaling by dephosphorylate and inactivate Akt1 and Akt3 [6]. Moreover, PHLPP has been found to act as a tumor suppressor in several types of cancer due to its ability to block growth factor-induced signaling in cancer cells [5,7]. FOXO1 belongs to subfamily of the forkhead transcription factors which are characterized by a conserved forkhead DNA binding domain [8]. It has been reported that FOXO transcription factors are involved in various signaling pathway and modulate a broad range of biochemical processes, such as cell cycle progression, differentiation, DNA damage repair, and apoptosis [8-11]. FOXO1 is known as a tumor suppressor, and deregulation of FOXO1 is involved in a variety of tumors [12-15]. As FOXO transcription factors are downstream targets of the serine/threonine protein kinase B (PKB)/Akt, the inhibition of FOXO1 is due to high level activation of Akt or other kinases. Activited Akt leads to FOXO1 phosphorylation, and results in FOXO1 translocating from nucleus to cytoplasm, and then being followed with ubiquitinatination and degradation. Since both PHLPP2 and FOXO1 are the negative regulators of Akt signaling pathway, the regulatory mechanism of these two regulators during bladder cancer progression arouses attention.

Multiple biomarkers have been identified, providing a better understanding of the molecular mechanisms involved in bladder carcinogenesis and tumor progression. MicroRNAs (miRNAs), a class of endogenous, small non-coding RNAs of 20–22 nucleotides, have been shown to be dysregulated in various human cancers and play important roles in cancer pathogenesis. MiRNAs function by regulating a variety of target genes, through binding with partially complementary sequences in the 3′-untranslated regions (3′-UTR) of targeted mRNAs [16,17]. Increasing numbers of reports indicate that miRNAs can act as diagnostic or prognostic markers of bladder cancer [18-20]. Furthermore, miRNAs are involved in the modulation of many biological processes of bladder cancers, including cell proliferation, apoptosis, cell invasion and migration, and angiogenesis [21-26].

In the present study, miR-135a was found to be upregulated in bladder cancer cells and tissues, indicating the potential function of miR-135a in bladder cancer development. Ectopic expression of miR-135a in bladder cancer cells led to the promotion of cell proliferation, tumorigenicity and cell cycle regulation, while inhibition of miR-135a suppressed the tumor proliferation and tumorigenesis. Moreover, our study presented data indicating that PHLPP2 and FOXO1 are direct targets of, and downregulated by, miR-135a. Thus, miR-135a plays essential role during the regulation of PHLPP2 and FOXO1, followed with activation of Akt related pathway in bladder cancer cells. Our present study suggested that miR-135a promotes cell proliferation, tumorigenicity and cell cycle progression in bladder cells by activating Akt signaling pathway via targeting PHLPP2 and FOXO1 mRNA and suppressing their expression.

## Materials and methods

### Cell culture and tissue specimens

Human bladder cancer cells (EJ, T24, BIU87, SCaBER, and 5637) were cultured in RPMI 1640 medium (Invitrogen, Carlsbad, CA, USA), supplemented with 10% fetal bovine serum (HyClone, Logan, UT, USA), penicillin and streptomycin (100 IU/ml) at 37°C under a 5% CO_2_ atmosphere in a humidified incubator. The primary cultures of normal bladder epithelial cells and bladder cancer cells established from fresh specimens of the normal bladder tissues and bladder cancer tissues respectively, had been histopathologically diagnosed and verified by experienced pathologists. The bladder biopsy was cut into two pieces; first piece would be used for establishing normal bladder epithelial cells. Second piece would be examined whether it is noncancerous bladder tissue by routine histopathological analysis, which confirms that the bladder epithelial cells established from above-mentioned first piece, are normal bladder epithelial cells (shown as NC). In brief, surgical specimens from normal bladder were promptly removed and transported aseptically in Hanks’ solution (Invitrogen) with 100 units/ml penicillin, and 100 μg/ml streptomycin (Invitrogen) and 5 μg/ml gentamicin (Invitrogen). The tissue specimens were incubated with 1.5 units/ml dispase (Roche Molecular Biochemicals) at 4°C overnight, and the epithelium was dissected away and incubated with trypsin (Invitrogen). The reaction was stopped with soybean trypsin inhibitor (Sigma, Saint Louis, MI) and centrifuged. The pellet was resuspended in keratinocyte-SFM medium (KSFM) (Invitrogen) supplemented with 40 μg/ml bovine pituitary extract (Invitrogen), 1.0 ng/ml EGF (Invitrogen), 100 units/ml penicillin, 100 μg/ml streptomycin (Invitrogen), 5 μg/ml gentamycin, and 100 units/ml nyastatin (Invitrogen). Normal bladder cells were grown at 37°C and 5% CO2 with KSFM, with 40 μg/ml bovine pituitary extract, 1.0 ng/ml EGF, 100 units/ml penicillin, and 100 μg/ml streptomycin) [27,28].

Bladder tumor tissues and adjacent non-tumor bladder tissues were collected from radical cystectomy and diagnosed histopathologically at the Department of Urology, the First Affiliated Hospital, Sun Yat-Sen University (the pathology of the tumor was: the sample #1, #2, #3 and #5 are of grade III, the sample #4 and #6 are of grade II, and the sample #7 is of grade I and all the samples were originated as urothelial cancers). None of patients underwent chemotherapy, radiotherapy or adjuvant treatment before surgery. All samples were obtained with written informed consent and approved by the Institutional Research Ethics Committee of the First Affiliated Hospital of Sun Yat-Sen University. The tissues and the matched adjacent non-cancerous bladder tissues were frozen and stored in liquid nitrogen for further use.

### Generation of stably engineered cell lines

The miR-135a expression plasmid, pMSCV-miR-135a, was generated by cloning the genomic pre-miR-135a gene into the retroviral transfer plasmid pMSCV-puro (Clontech Laboratories Inc., Mountain View, CA, USA). Stable cell lines expressing miR-135a were established via retroviral infection according to a previously reported protocol [29] and stably transduced cells were selected by treatment with 1.5 μg/ml puromycin for 10 days beginning at 48 h after infection. The expression level of stably engineered cell lines was examined by real-time PCR.

### RNA extraction, reverse transcription (RT) and real-time PCR

Total cellular RNA was extracted using TRIzol reagent (Invitrogen, Carlsbad, CA, USA) according to the protocol provided by the manufacturer and used to synthesize cDNA with specific stem-loop primers and the TaqMan MicroRNA Reverse Transcription Kit (Applied Biosystems; Foster City, CA, USA). Expression of miRNAs was analyzed using the TaqMan MicroRNA Assay kit (Applied Biosystems). Gene expression levels were quantified using the 7500 Fast Real-Time Sequence detection system software (Applied Biosystems). Gene expression was defined based on the threshold cycle (Ct) normalized to that of the housekeeping gene *GAPDH* as the control (GAPDH forward primer, 5′-GACTCATGACCACAGTCCATGC-3′; reverse primer, 3′-AGAGGCAGGGATGAT GTTCTG-5′), and calculated as 2^-[(Ct^^of p21, p27, CyclinD1, Ki67) – (Ct^^of GAPDH)]^. The relative expression levels of miRNA were calculated as 2^-[(Ct of miR-135a) – (Ct of U6)]^. Small nuclear RNA U6 was used for normalization, and the primers for miRNA were purchased from RiboBio (RiboBio Co. Ltd, Guangzhou, Guangdong, China).

PCR amplification of genes was carried out using the following thermal conditions: 95°C for 30 s followed by 40 cycles of 95°C for 5 s and 60°C for 30 s. The PCR conditions for amplification of miR-135a were: 95°C for 20 s, followed by 40 cycles of 95°C for 10 s and 60°C for 20 s, with a final incubation at 70°C for 5 s.

The primers selected were as follows:

- p21 forward: 5′-CATGGGTTCTGACGGACAT -3′,
- p21 reverse: 5′- AGTCAGTTCCTTGTGGAGCC -3′;
- p27 forward: 5′- TGCAACCGACGATTCTTCTACTCAA -3′,
- p27 reverse: 5′- CAAGCAGTGATGTATCTGATAAACAAGGA-3′.
- Cyclin D1 forward: 5′-AACTACCTGGACCGCTTCCT -3′,
- Cyclin D1 reverse: 5′-CCACTT GAGCTTGTTCACCA-3′.
- Ki67 forward: 5′- AGGACTTTGTGCTCTGTAACC -3′,
- Ki67 reverse: 5′- CTCTTTTGGCTTCCATTTCTTC -3′.

### Western blotting assay

Total protein was extracted from whole cells by a previously described procedure. Lysate protein (20 μg) for Western blotting was separated by SDS-PAGE and electroblotted onto a PVDF membrane (Bio-Rad Laboratories, Hercules, CA, USA). The membranes were probed with polyclonal rabbit antibodies against anti-FOXO1, anti-p21, anti-p27, anti-CyclinD1, and anti-Ki67 (1:1,000; Cell Signaling, Danvers, MA, USA). The membranes were stripped and re-probed with an anti-α-tubulin mouse monoclonal antibody (1:1,000; Cell Signaling) as a loading control.

### Oligonucleotides, siRNA and transfection

The miR-135a mimic (50 nM), miR-135a inhibitor (100 nM; miR-inhibitor is a commercial product from a professional bio-company “RiboBio” (RiboBio Co. Ltd, Guangzhou, Guangdong, China), is a LNA/OMe modified antisense oligonucleotide designed specifically to bind to and inhibit endogenous miR molecule with rarely off-target effect), and negative control (NC) were purchased from RiboBio. For depletion of FOXO1, the siRNA (50 nM) was synthesized and purified by RiboBio. The PHLPP2 siRNA sequence: 5′-CCTAAGTGGCAACAAGCTT-3′; FOXO1 siRNA sequence used were: 5′-GCA AAGAUGGCCUCUACUU-3′; Transfections with oligonucleotides and siRNA were performed using the Lipofectamine 2000 reagent (Invitrogen) according to the manufacturer’s instructions.

### Cell viability assay

Cells were seeded in 96-well plates at 2 × 10^3^ per well. At the indicated time-points, 100 μl MTT (0.5 mg/ml, Sigma, Saint Louis, MO, USA) was added to each well and cells were incubated for 4 h at 37°C, followed by removal of the culture medium and addition of 150 μl DMSO (Sigma). The absorbance was measured at 570 nm, with 655 nm as the reference wavelength. All experiments were performed in triplicate.

### Colony formation assay

Cells (0.5 × 10^3^ cells per well) were seeded into 6-well plates and cultured for 14 days. Cells were subsequently fixed with glutaraldehyde (6.0% v/v, Sigma), and stained with crystal violet (0.5% w/v, Sigma) for 5 min. The number of colonies formed was counted in 10 different fields of vision and the mean value was calculated. The experiment was performed independently three times for each cell line.

### Anchorage-independent growth ability assay

An agar layer, comprising a 0.6% complete medium agar mixture, was prepared in a 6 cm tissue culture dish. Subsequently, 1.5 × 10^3^ cells, suspended in 2 ml complete medium plus 0.3% agar (Sigma), were plated on top of the agar layer. The cells were incubated at 37°C for 2 weeks to allow for growth of colonies. Colonies greater than 0.1 mm in diameter were counted. The experiment was performed independently three times for each cell line.

### Flow cytometric cell cycle analysis

Cells (1 × 10^6^) were harvested and fixed in 75% ice-cold ethanol. Before cell cycle analysis, cells were treated with bovine pancreatic RNase (2 μg/ml; Sigma) at 37°C for 30 min, followed by incubation in propidium iodide (20 μg/ml; Sigma) for 20 min. Cell cycle analysis was performed using the BD LSRII Flow Cytometry System with FACSDiva software (BD Bioscience, Franklin Lakes, USA). The data were analyzed with the ModFit LT software package and the cell cycle distribution was shown as the percentage of cells in the G1, S, and G2 populations.

### Bromodeoxyuridine labeling and immunofluorescence

Cells (7 × 10^4^) grown on coverslips were incubated with bromodeoxyuridine (BrdUrd) for 1 h and stained with anti-BrdUrd antibody (Upstate, Temecula, CA) according to the manufacturer’s instructions. Gray level images were acquired under a laser scanning microscope (Axioskop 2 plus, Carl Zeiss Co. Ltd., Jena, Germany).

### Luciferase assay

The luciferase miRNA target expression vector pGL3 was used for 3′-UTR luciferase assays (Promega, Madison, WI, USA). The primer sequences for the wild-type 3′UTR (334 bp) were: Forward, 5′-CGGGGTACCAGTTGGCCTCTCCTTGAGGT 3′; Reverse, 5′-GAAGATCTGGCTGACAAGACTTAACTCAA-3′. For the mutant 3′UTR, QuikChange® Site-Directed Mutagenesis Kit (Agilent Technologies, Inc., CA, USA) was used and the primer sequences were: Forward, 5′-CAATCTTTGCTATAATTGTATAA AG**GGT**TAAATGTACATAAATTATGTTTAAATGGCTTG-3′; Reverse, 5′-CAAGCCA TTTAAACATAATTTATGTACATTTA**ACC**CTTTATACAATTATAGCAAAGATTG-3′. For luciferase assays, cells (5 × 10^4^) were plated in a 24-well plate and incubated for 24 h prior to transfection. Firefly luciferase constructs containing the 3′UTR (or 3′UTR-mutant) of the potential miR-135a target (100 ng), pRL-TK Renilla luciferase normalization control (1 ng; Promega), miRNA mimic, inhibitor or negative control were cotransfected using Lipofectamine 2000 (Invitrogen). Lysates were collected 48 h after transfection and measured using a Dual-Luciferase Reporter System (Promega) according to the manufacturer protocol. Three independent experiments were performed and the data were presented as the mean ± SD.

### Statistical analysis

Student’s *t*-test (using a two-tailed paired *t*-test) was used to evaluate the significant difference of two groups of data in all the pertinent experiments. All data were expressed as the mean ± standard deviation (SD) for three independent experiments. A *P-*value < 0.05 was regarded as statistically significant.

## Results

### MiR-135a expression is elevated in bladder cancer

**Figure 1:**
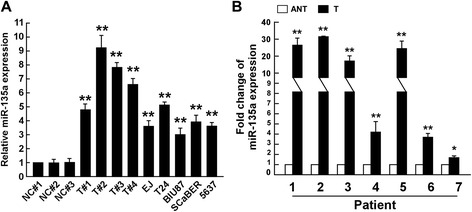
**Expression of miR-135a is elevated in bladder cancer cells. A**. Real-time PCR analysis of miR-135a expression in bladder cancer cells, including primary culture cells of bladder cancer tissues (shown as T#1, T#2, T#3) and bladder cancer cell lines, EJ, T24, BIU87, SCaBER, and 5637, compared to normal bladder cells as controls. The normal control for bladder cancer cell lines are primary cultures of normal bladder epithelial cells established from fresh specimens of normal bladder tissues (shown as NC #1, #2, #3). **B**. The expression of miR-135a was examined in seven pairs of cancerous tissues (T) and their adjacent non-cancerous bladder tissues (ANT). The average miR-135a expression was normalized using U6 expression. Bars represent the mean ± SD of three independent experiments. **P <0.01, *P <0.05.

the expression of miR-135a was exmined by real-time RT-PCR in bladder cancer. MiR-135a was found to be markedly upregulated in bladder cancer cells, including primary cultures of bladder cancer tissues and bladder cancer cell lines, EJ, T24, BIU87, SCaBER, and 5637, compared with normal bladder epithelial cells (primary cultures of normal bladder tissues) (Figure 1A). In order to further verify the expression pattern of miR-135a in human bladder cancer tissues, we examined and compared the expression levels of miR-135a in seven pairs of human bladder cancer tissues and adjacent non-tumor tissues. The results showed that the relative expression of miR-135a in bladder cancer tissues was significantly higher than that of their matched adjacent normal tissues (n = 7, r = 0.896, *P* = 0.006; Figure 1B). These data suggested that miR-135a expression was elevated in bladder cancer, indicating a putative tumor-promoter function of miR-135a in bladder cancer.

### MiR-135a promotes bladder cancer cell proliferation and cell cycle progression

**Figure 2:**
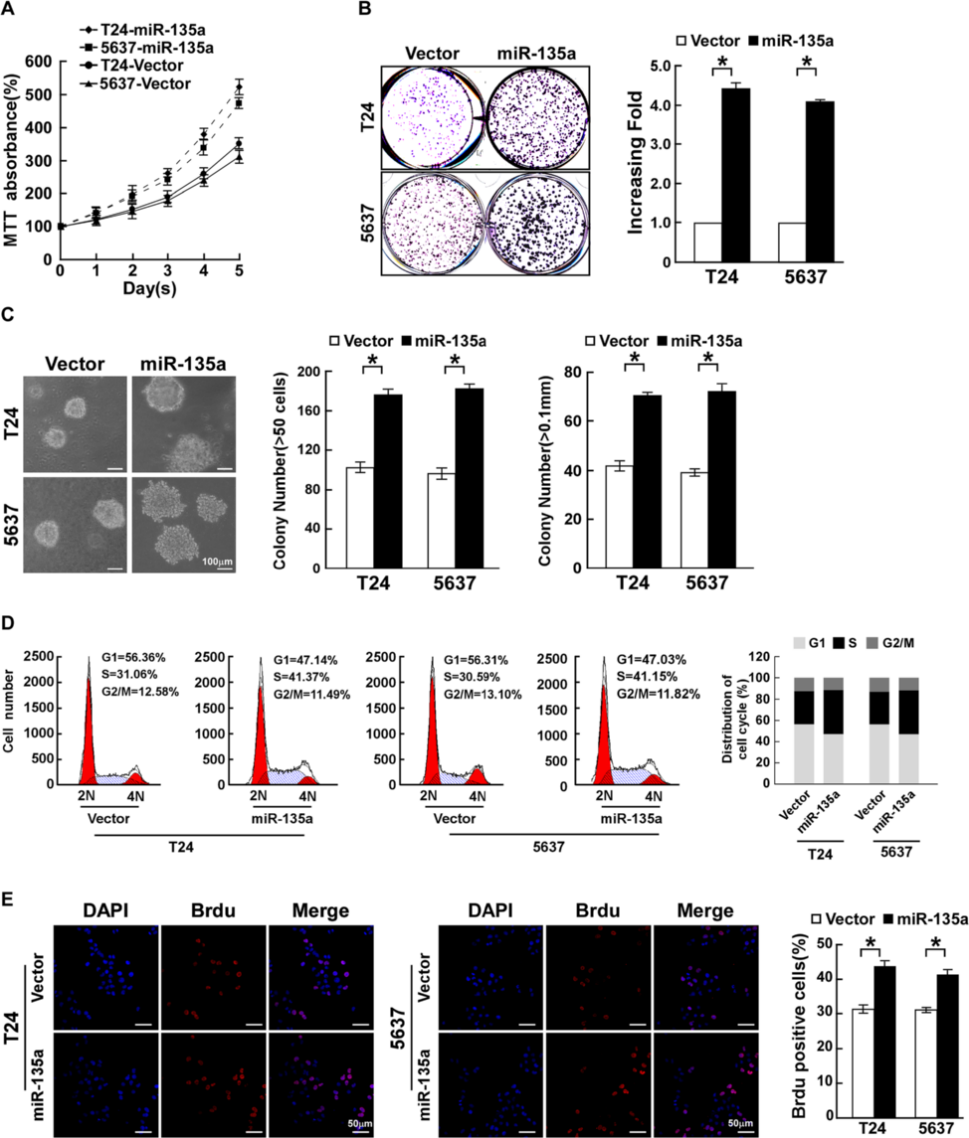
**MiR-135a induces proliferation of bladder cancer cells. A**. Effects of miR-135a on proliferation of the indicated cells, as analyzed by MTT assays. **B**. Representative micrographs (left) and quantifications (right) of crystal violet stained colonies formed by the indicated cells. **C**. Effects of ectopic miR-135a expression on the tumorigenicity of the indicated cells, as determined by anchorage-independent growth ability assays. **D**. Effects of ectopic miR-135a expression on cell cycle progression of the indicated cells, as analyzed by flow cytometry. **E**. Representative micrographs (left) and quantification (right) of the BrdUrd incorporation assay in the indicated cells. All these experiments were done with T24 and 5637 cells stably overexpressing miR-135a or pMSCV-vector. Bars represent the mean ± SD of three independent experiments. *P <0.05.

To determine the tumor-promoter function of miR-135a in bladder cancer progression, T24, 5637 and BIU87 cells stably overexpressing miR-135a were established for further investigations. An MTT assay showed that ectopic expression of miR-135a significantly increased the growth rate of bladder cells (Figure 2A, Additional file 1: Figure S1A). Meanwhile, colony formation assays showed that overexpression of miR-135a enhanced the proliferation of bladder cancer cells, and colonies were much larger in bladder cancer cells stably overexpressing miR-135a (Figure 2B, Additional file 1: Figure S1B and Additional file 2: Figure S2A). Additionally, an anchorage-independent growth assay revealed that bladder cells stably overexpressing miR-135a showed more and larger-sized colonies than control cells (Figure 2C, Additional file 1: Figure S1C and Additional file 2: Figure S2B). The cell cycle analysis of T24 and 5637 cells by flow cytometry showed a significant decrease in the percentage of cells in G1/G0 phase and an increase in the percentage of cells in S phase (Figure 2D). The level of DNA synthesis, when examined by BrdUrd incorporation assay, was significantly elevated in miR-135a overexpressing T24 and 5637 cells, whereas the control cells displayed relatively lower BrdUrd incorporation rates (Figure 2E). These results indicated that overexpression of miR-135a promoted the proliferation, tumorigenicity and cell cycle progression of bladder cancer cells *in vitro*.

### Inhibition of miR-135a suppresses proliferative capacity of bladder cancer cells

**Figure 3:**
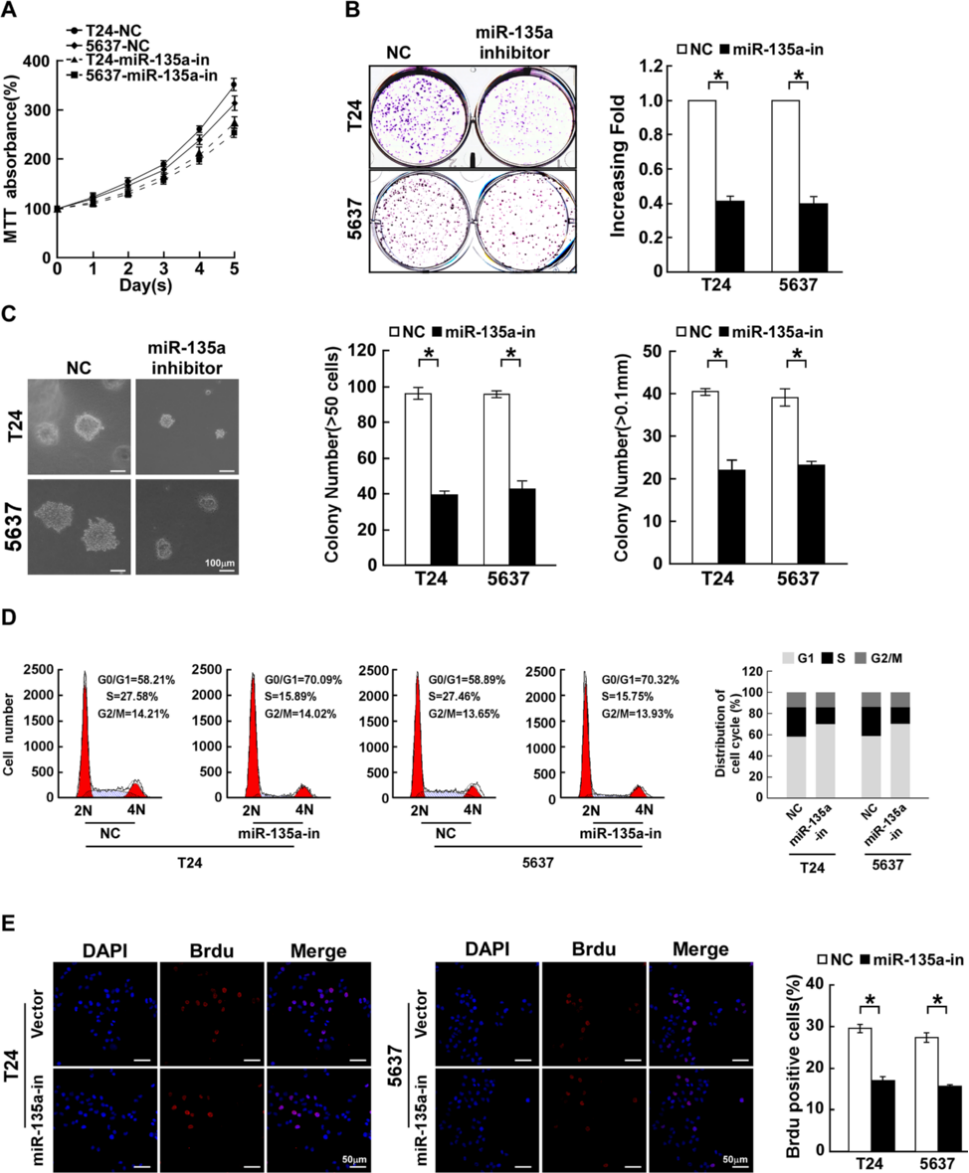
**Inhibition of miR-135a suppresses the growth of bladder cancer cells. A**. Effects of miR-135a inhibitor (shown as miR-135a-in) or negative control (shown as NC) on the proliferation of bladder cancer cells, as analyzed by MTT assays. **B**. Representative micrographs (left) and quantifications (right) of crystal violet stained colonies formed by the indicated cells. **C**. Effects of miR-135a inhibitor or negative control on the tumorigenicity of the indicated cells, as determined by anchorage-independent growth ability assays. **D**. Effects of miR-135a inhibitor or negative control on cell cycle progression of the indicated cells, as analyzed by flow cytometry. **E**. Representative micrographs (left) and quantification (right) of the BrdUrd incorporation assay in the indicated cells. Bars represent the mean ± SD of three independent experiments. *P <0.05.

Loss-of-function studies using a miR-135a inhibitor were performed to further confirm whether inhibition of miR-135a reduces bladder cancer cell proliferation. MTT and colony formation assays showed that suppression of miR-135a significantly decreased the proliferative capacity of T24 and 5637 cells transfected with the miR-135a inhibitor compared with that of NC transfected cells (Figure 3A and B). The anchorage-independent growth assay revealed that suppression of miR-135a led to fewer and smaller colonies than the control cells (Figure 3C). Flow cytometry showed a significant increase in the percentage of cells in G1/G0 phase and a decrease in the percentage of cells in S phase in cells transfected with the miR-135a inhibitor compared with NC transfected cells (Figure 3D). BrdUrd incorporation assays showed that the level of DNA synthesis was reduced by inhibition of miR-135a (Figure 3E). These results suggested that downregulation of miR-135a reduces the proliferation and tumorigenicity of bladder cancer cells.

### MiR-135a decreases expression of the cell cycle inhibitors p21^Cip1^ and p27^Kip1^ and increases expression of cell cycle regulator Cyclin D1

**Figure 4:**
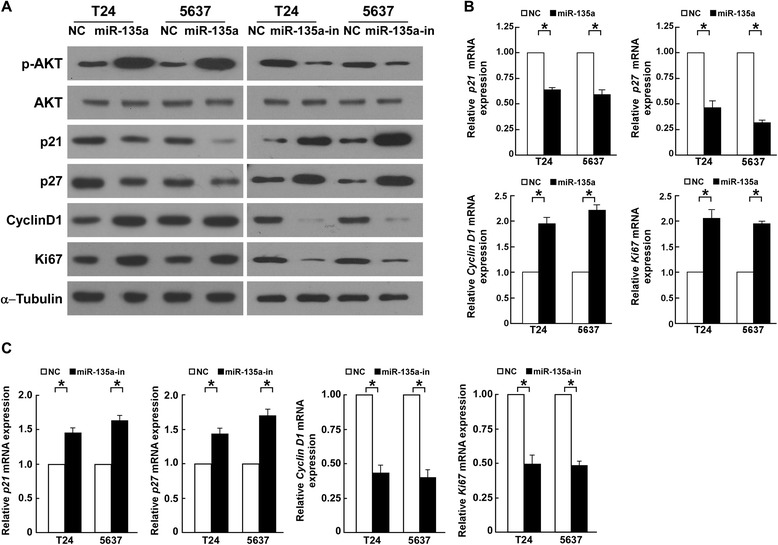
**MiR-135a modulates expression of cell cycle regulators, including p21** ^**Cip1**^ **, p27** ^**Kip1**^ **, Cyclin D1 and Ki67. A**. Western blotting analysis of p21^Cip1^, p27^Kip1^ , Cyclin D1 and Ki67 in indicated bladder cancer cells. α-Tubulin was used as a loading control. **B**. Real-time PCR analysis of *p21* ^*Cip1*^, *p27* ^*Kip1*^, *Cyclin D1* and *Ki67* mRNA in bladder cancer cells transfected with miR-135a or NC. **C**. Real-time PCR analysis of *p21* ^*Cip1*^ *, p27* ^*Kip1*^, *Cyclin D1* and *Ki67* mRNA in bladder cancer cells transfected with the miR-135a inhibitor or NC. *GAPDH* was used as a loading control. Bars represent the mean ± SD values of three independent experiments; *P < 0.05.

As miR-135a promoted cell proliferation, we further investigated the effect of miR-135a on the expression of the genes which regulate cell cycle and proliferation, including p21^Cip1^, p27^Kip1^ and Cyclin D1. Compared to NC transfected cells, p21^Cip1^ and p27^Kip1^ were downregulated and Cyclin D1 was upregulated in miR-135a-transfected cells, while p21^Cip1^ and p27^Kip1^ were upregulated and Cyclin D1 was downregulated in miR-135a-inhibitor-transfected cells (Figure 4A–C). Coincident with altered expression of cell cycle regulators, the expression of Ki67, a cellular marker for proliferation, was also significantly increased in miR-135a-transfected cells, and decreased in miR-135a-inhibitor-transfected cells (Figure 4A–C), which further confirmed that miR-135a promoted the proliferation and cell cycle progression of bladder cancer cells.

### PHLPP2 and FOXO1 are direct targets of miR-135a in bladder cancer cells

**Figure 5:**
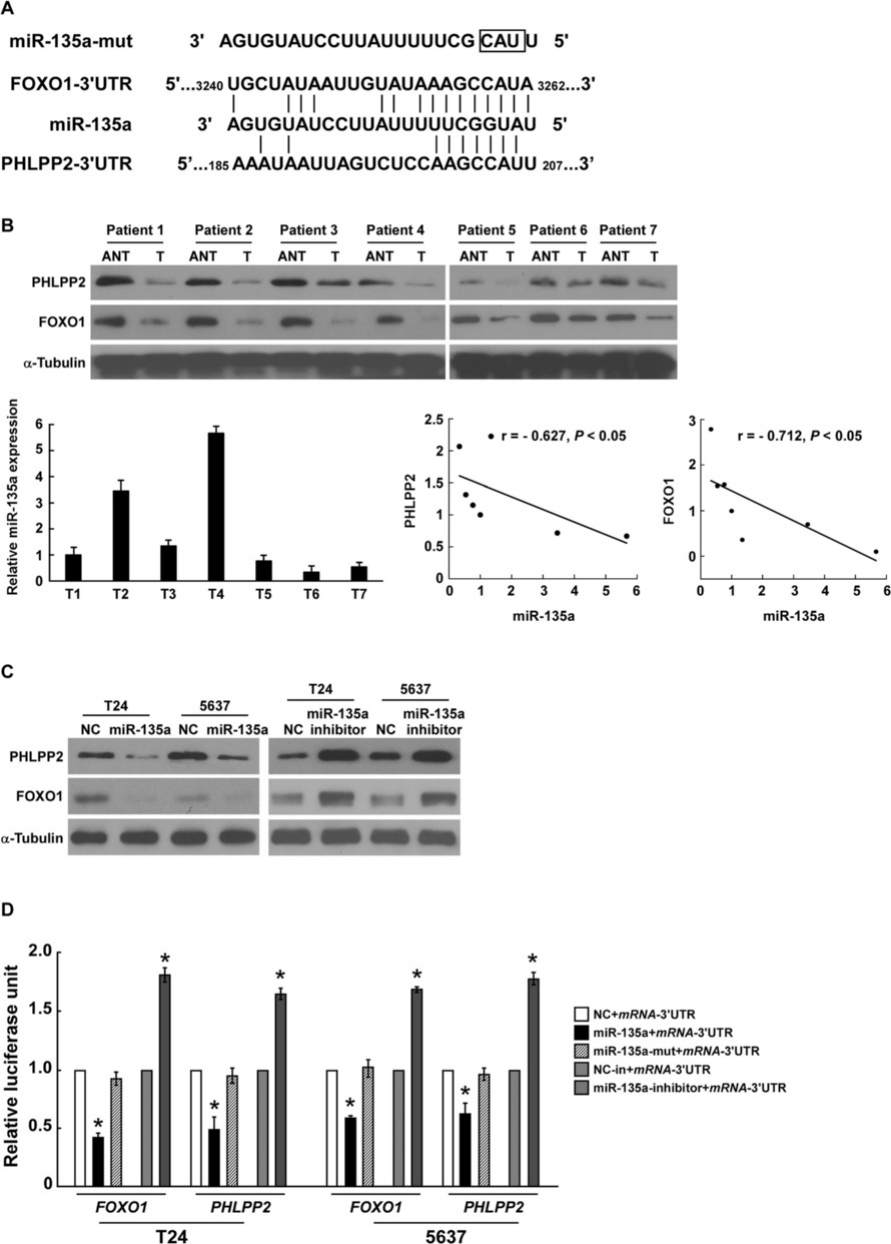
**PHLPP2 and FOXO1 are potential targets of miR-135a. A**. Sequence of *PHLPP2*- or *FOXO1*-3′UTR miR-135a binding seed region, miR-135 and mutation of miR-135a (shown as miR-135a-mut). **B**. Upper: Western blotting analysis of PHLPP2 or FOXO1 in bladder cancer tissues, compared with normal bladder tissue. α-Tubulin was used as a loading control. Lower: Real-time PCR analysis of miR-135a in bladder cancer tissues, and the correlation between miR-135a and PHLPP2 or FOXO1 expression in bladder cancer tissues. **C**. The expression level of PHLPP2 or FOXO1 protein in bladder cancer cells transfected with miR-135a or miR-135a inhibitor, respectively, compared with control cells (shown as NC), by Western blotting analysis. α-Tubulin was used as a loading control. **D**. PHLPP2 or FOXO1 luciferase reporter activity in the indicated cells, measured by luciferase assay. pRL-TK Renilla luciferase was used as the normalization control; Bars represents the mean ± SD of three independent experiments. *P <0.05.

To explore the molecular mechanism of miR-135a function in bladder cancer cells, the target of miR-135a was predicted using the publicly available algorithms (TargetScan). PHLPP2 and FOXO1 were identified among the putative targets of miR-135a and the potential binding sequence for miR-135a was identified within the 3′UTR of PHLPP2 and FOXO1 (Figure 5A). The protein expression of PHLPP2 and FOXO1 were further found to be decreased in the bladder cancer tissues, compared with that of the normal bladder tissue, and miR-135a expression in these bladder cancer tissues was inversely correlated with the expression of PHLPP2 (r = −0.627, *P* < 0.05) and FOXO1 (r =-0.712, *P* < 0.05) (Figure 5B). Meanwhile, cells overexpressing miR-135a exhibited significantly lower expression of PHLPP2 and FOXO1, while inhibition of miR-135a led to higher PHLPP2 and FOXO1 expression (Figure 5C). Co-transfection of miR-135a with the pGL3-PHLPP2-3′UTR or pGL3-FOXO1-3′UTR luciferase reporter plasmid caused a remarkable decrease of luciferase activity, whereas inhibition of miR-135a led to increased luciferase activity (Figure 5D). However, luciferase activity was not affected by miR-135a-mut transfection instead of miR-135a;or pGL3-PHLPP2/FOXO1-3′UTR mutant instead of pGL3-PHLPP2/FOXO1-3′UTR (Figure 5A and D; Additional file 3: Figure S3). Collectively, these results suggested that miR-135a directly targets FOXO1 in bladder cancer cells.

### Suppression of PHLPP2 or FOXO1 is essential for miR-135a-induced cell proliferation in bladder cancer

**Figure 6:**
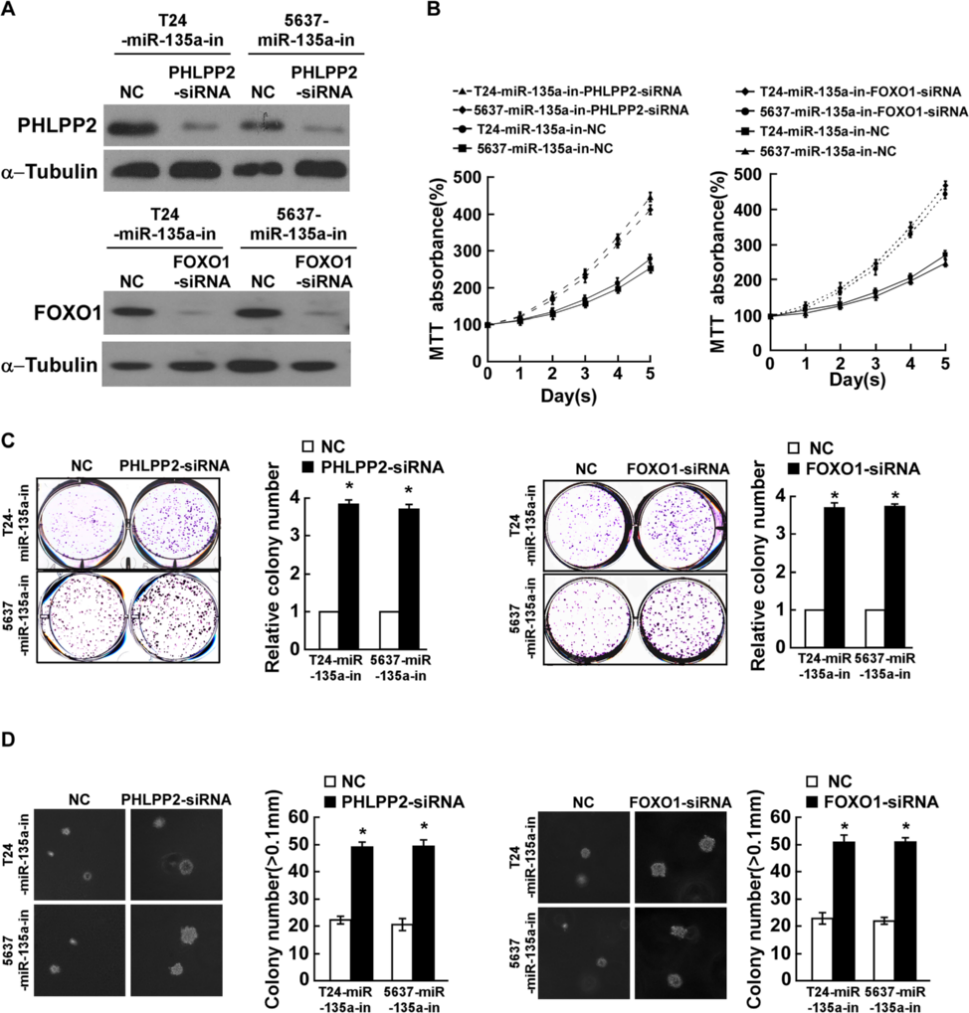
**MiR-135a promotes proliferation of bladder cancer cells by inhibiting PHLPP2 and FOXO1. A**. The expression level of PHLPP2 or FOXO1 in miR-135a-inhibitor transfected bladder cells that were transfected with PHLPP2- or FOXO1-siRNA, measured by Western blotting assays; α-Tubulin served as the loading control. **B**. The growth rates in PHLPP2- or FOXO-silenced cells, measured by MTT assays. **C**. Representative micrographs (left) and quantifications (right) of crystal violet stained colonies formed by the indicated cells. **D**. Representative images (left) and quantifications (right) of colonies formed by the indicated cells determined by anchorage-independent growth assay. Bars represent the mean ± SD from three independent experiments. *P < 0.05.

To further confirm the importance effect of PHLPP2 or FOXO1 suppression during miR-135a-induced bladder cancer proliferation, a specific siRNA of PHLPP2 or FOXO1 was used to suppress endogenous PHLPP2 or FOXO1 expression, respectively (Figure 6A). The results of cell viability MTT and the colony formation assay both indicated that suppression of PHLPP2 or FOXO1 in cells transfected with miR-135a inhibitor dramatically increased the proliferation of bladder cancer cells (Figure 6B and C). Furthermore, the anchorage-independent growth assay showed the similar results (Figure 6D). These results suggested that silencing PHLPP2 and FOXO1 expression in miR-135a-repressed cells could reverse the inhibitory effect of the miR-135a inhibitor on bladder cancer cells proliferation. It is confirmed that miR-135a promotes bladder cancer cells proliferation and tumorigenicity by suppressing endogenous PHLPP2 and FOXO1 expression, and that PHLPP2 and FOXO1 suppression are essential for miR-135a-mediated cell proliferation in bladder cancer.

## Discussion

**Figure 7:**
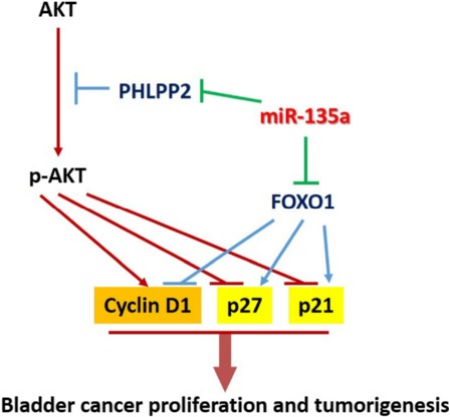
**The model of miR-135a-mediated Akt signaling activation through down-regulation of PHLPP2 and FOXO1 that results in the promotion of bladder cancer cell proliferation and tumorigenesis.**

Recently, the roles of miRNAs in the regulation of tumor progression and development are attracting increasing attention, with growing numbers of reports suggesting that dysregulation of miRNAs contributes to certain types of tumor progression. In the present study, we found that miR-135a is significantly upregulated in bladder cancer cells and tissue, leading to increased bladder cell proliferation and tumorigenicity. To explore the mechanism of miR-135a-induced cell proliferation, we investigated potential targets of miR-135a and found that PHLPP2 and FOXO1 are directly targeted by miR-135a and are essential for the bio-function of miR-135a in bladder cancer. The negative regulation of PHLPP2 and FOXO1 by miR-135a leads to dysregulation of phosphorylated Akt, p21, p27, CyclinD1 and Ki67, indicating that miR-135a plays an essential role in bladder cancer progression through Akt-mediated signaling (Figure 7).

As bladder cancer is the most common malignancy involving the urinary system, much research has been focused on the biological properties and progression of bladder cancer. Based on the investigations of the biological and predictive functions of miRNAs in malignancies, data are emerging that elucidate the effects of miRNAs in bladder cancer. MiR-409-3p is reported to be downregulated in bladder cancer and ectopic miR-409-3p expression significantly reduced bladder cancer cell migration and invasion [30]. MiR-23b was found to act as a tumor suppressor in bladder cancer cells and overexpression of miR-23b inhibited cell proliferation and colony formation. Furthermore, miR-23b expression is repressed in bladder cancer and is considered to be a potential diagnostic and prognostic biomarker, with high miR-23b expression being positively correlated with higher overall survival of bladder cancer patients [31]. Liu *et al*. demonstrated that miR-135a contributes to the development of portal vein tumor thrombus by promoting invasion and metastasis in hepatocellular carcinoma [32]. A miRNA-135a/b binding polymorphism, the SNP rs2240688A > C in the 3′-untranslated region of CD133, is considered to be a functional biomarker for the prediction of risk and prognosis in lung cancer. It is reported this SNP decreases risk and favorable prognosis in lung cancer by miRNA-135a/b-mediated reduction of CD133 expression [33]. Moreover, miR-135a has been reported as a tumor-suppressive factor that inhibits cancer cell proliferation by targeting c-MYC in renal cell carcinoma [34]. All the previous studies indicated that the function of miR-135a in tumor progression is complicated and segmentary, which needs further investigation. The biological function of miR-135a and its mechanism in bladder cancer development has not been elucidated. Our study reveals for the first time that miR-135a acts as an onco-miR and promotes cell proliferation by reducing FOXO1 expression in bladder cancer.

As has been reported, miRNAs play essential roles in translational repression by targeting the 3′ untranslated region (3′-UTR) of mRNAs in a sequence-specific manner. MiR-490-5p inhibits proliferation of bladder cancer cells by targeting c-Fos [21]. MiR-16 inhibits bladder cancer proliferation by targeting Cyclin D1 [22]. C-Met is a direct target of miR-409-3p and mediates the biological function of miR-409–3p in bladder cancer [30]. MiR-23b inhibits cell proliferation by post-transcriptionally regulating Zeb1 in bladder cancer [31]. It has been reported that MiR-135a promotes growth and invasion of colorectal cancer via targeting and downregulating metastasis suppressor 1, which is absent or reduced in cancer cells [35]. Wu *et al.* found miR-135a targets JAK2 and reduces p-STAT3 activation and cyclin D1 and Bcl-xL protein expression, leading to inhibition of gastric cancer cell proliferation [36]. Moreover, miR-135a is involved in colorectal cancer pathogenesis via targeting and suppressing adenomatous polyposis coli (APC) and inducing downstream Wnt pathway activity [37]. In addition, miRNA-135a was also found to promote breast cancer cell migration and invasion by targeting HOXA10 [38]. However, to date, the exact functions and regulatory mechanisms of miR-135a in tumorigenesis of bladder cancer remain largely unknown. Currently, we demonstrate that PHLPP2 and FOXO1 are the direct targets of miR-135a and that silencing PHLPP2 or FOXO1 plays a crucial role in miR-135a-induced proliferation of bladder cancer cells. In addition, as there are various targets that correlate with multiple biological function of miR-135a in other tumors confirmed by previous studies, the potential function of miR-135a on other bladder cancer biology might be of great interest to explore.

PHLPP2 has been found function as tumor suppressor in variety of malignancies. Loss of PHLPP expression, either PHLPP1 or PHLPP2, in colorectal cancer was found and overexpression of PHLPP inhibits proliferation of colon cancer both in vitro and in vivo [39]. It has been reported PHLPP2 is downregulated by miR-205 and leads to stimulation of proliferation and angiogenesis in non-small cell lung cancer cells [40]. Mei *et al*. found that repression PHLPP2 plays crucial roles in miR-141-induced proliferation of NSCLC cells [41]. However, the expression pattern and the regulatory mechanism of PHLPP2 in bladder cancer remains unfolded. Herein, in our present study, for the first time PHLPP2 was found to be downregulated in bladder cancer and miR-135a was found to target and repress the expression of PHLPP2, resulting the promotion of bladder cancer proliferation and tumorigenesis. FOXO1, a member of the forkhead box O (FOXO) subfamily of transcription factors, functions as a tumor suppressor and regulates genes involved in the apoptotic response, cell cycle checkpoints and cellular metabolism. Accumulating data suggest that FOXO1 is downregulated in various types of cancers. However, the molecular mechanism resulting in aberrant expression of FOXO1 is poorly understood. Recent evidence suggests that post-transcriptional regulation is important for FOXO1 downregulation and the modulation of its activity. It has been revealed that miR-96 plays an anti-apoptotic function in bladder and prostate cancer through suppression of FOXO1 expression by targeting its 3′UTR [23,42].

Moreover, PHLPP2 is found to be the suppressive regulator of Akt signaling pathway [5-7] and FOXO1 is known to be closely correlated with the phosphotidylinositide-3-kinase (PI3K)/AKT signaling pathway [14,15,43]. Further, activation of the PI3K/AKT signaling pathway leads to FOXO1 phosphorylation, which results in FOXO1 proteasomal degradation, and dysregulation of Cyclin D1, Cyclin D2, and p21 levels and Cyclin-Dependent Kinase-4 activity [43-45]. Consist with previous studies, our results suggested that PHLPP2 and FOXO1 are both downregulated by miR-135a in bladder cancer cells, and mediated miR-135a-induced cell proliferation. In addition, p21, p27, Cyclin D1 and Ki67 were found to be dysregulated in miR-135a- overexpressing bladder cancer cells, indicating a putative correlation between miR-135a and the PI3K/Akt signaling pathway. Further studies are required to elucidate the detailed molecular mechanism underlying the role of miR-135a in tumor progression.

## Conclusion

This study shows that miR-135a is significantly upregulated and promotes cellular proliferation in bladder cancer. The tumor suppressor genes, PHLPP2 and FOXO1, are the direct targets of miR-135a and transcriptionally downregulated by miR-135a. Suppression of PHLPP2 or FOXO1, followed with dysregulation of p21, p27, CyclinD1 and Ki67, play critical roles during miR-135a-mediated bladder cancer cells proliferation. MiR-135a functions as an onco-miR in bladder cancer and interventions targeting miR-135a may provide novel insights into approaches to bladder cancer diagnosis and therapy.

## Additional files

Additional file 1: Figure S1. MiR-135a induces proliferation of BIU87 cells. A. Effects of miR-135a on proliferation of the indicated cells, as analyzed by MTT assays. B. Representative micrographs (left) and quantifications (right) of crystal violet stained colonies formed by the indicated cells. C. Effects of miR-135a on the tumorigenicity of the indicated cells, as determined by anchorage-independent growth ability assays. Bars represent the mean ± SD of three independent experiments. **P* <0.05. Additional file 2: Figure S2. MiR-135a induces proliferation of bladder cancer cells. A. Quantifications of crystal violet stained colonies formed by the indicated cells. B. Effects of miR-135a on the tumorigenicity of the indicated cells, as determined by anchorage-independent growth ability assays. All these experiments were done with T24 and 5637 cells stably overexpressing miR-135a, pMSCV-vector (presented as Vector) or non-transfected cell control (presented as Blk). Bars represent the mean ± SD of three independent experiments. **P* <0.05. Additional file 3: Figure S3. FOXO1 and PHLPP2 are direct targets of miR-135a in bladder cancer cells. (A) Sequence of pGL3-FOXO1 or PHLPP2-3′UTR reporter with a mutant miR-135a binding site. (B) Luciferase assay of co-transfected with miR-135a or the control in the indicated cells. Experiments were repeated at least 3 times with similar results, and error bars represent ± SD, *P<0.05.
