# Supplementary figures and images for: Mir-135a enhances cellular proliferation through post-transcriptionally regulating PHLPP2 and FOXO1 in human bladder cancer
Source: J Transl Med. 2015 Mar 13;13:86. doi: 10.1186/s12967-015-0438-8 (PMC4367980; doi:10.1186/s12967-015-0438-8)

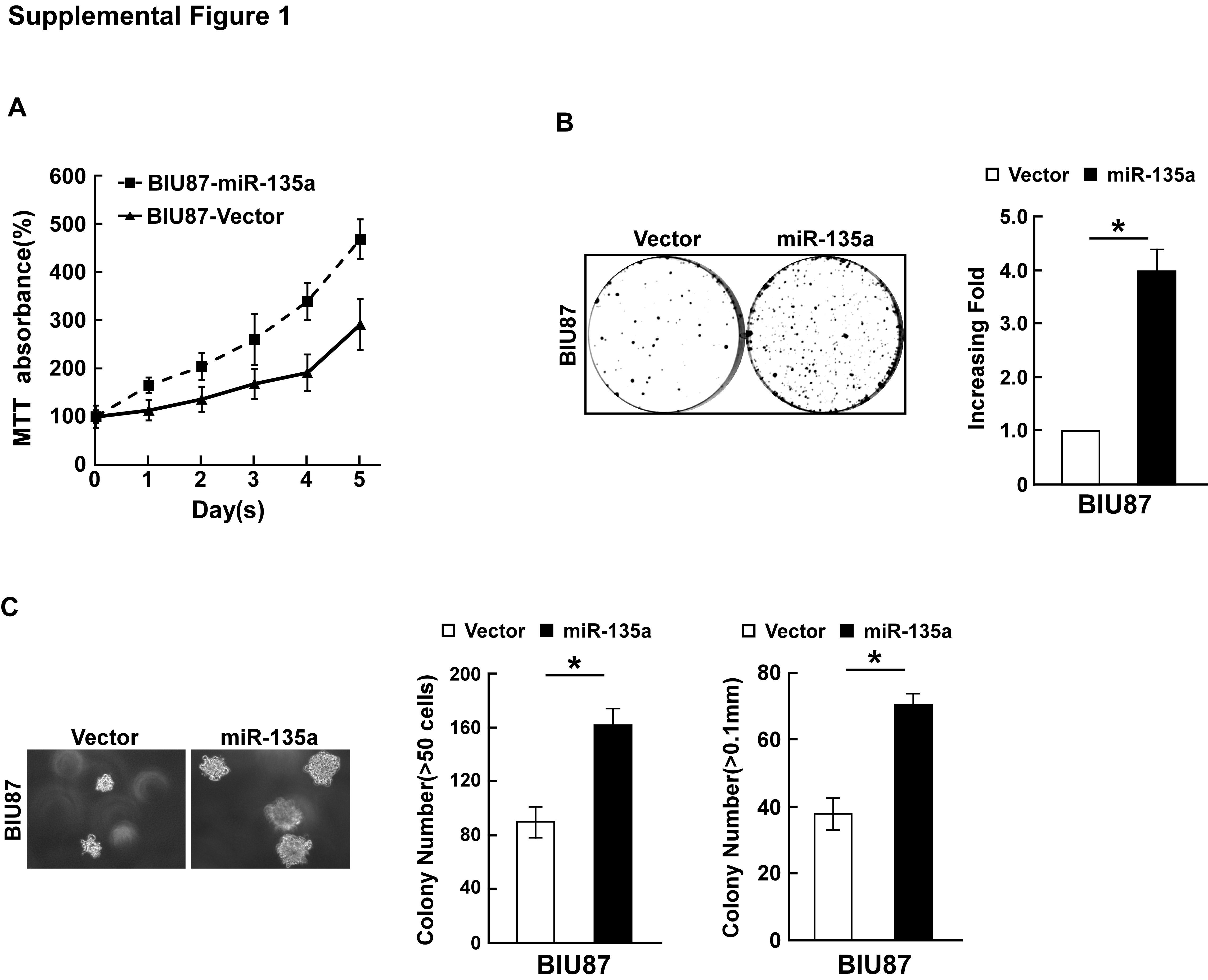

Supplement: Additional file 1: Figure S1. — MiR-135a induces proliferation of BIU87 cells. A. Effects of miR-135a on proliferation of the indicated cells, as analyzed by MTT assays. B. Representative micrographs (left) and quantifications (right) of crystal violet stained colonies formed by the indicated cells. C. Effects of miR-135a on the tumorigenicity of the indicated cells, as determined by anchorage-independent growth ability assays. Bars represent the mean ± SD of three independent experiments. *P <0.05. [file 12967_2015_438_MOESM1_ESM.tiff]

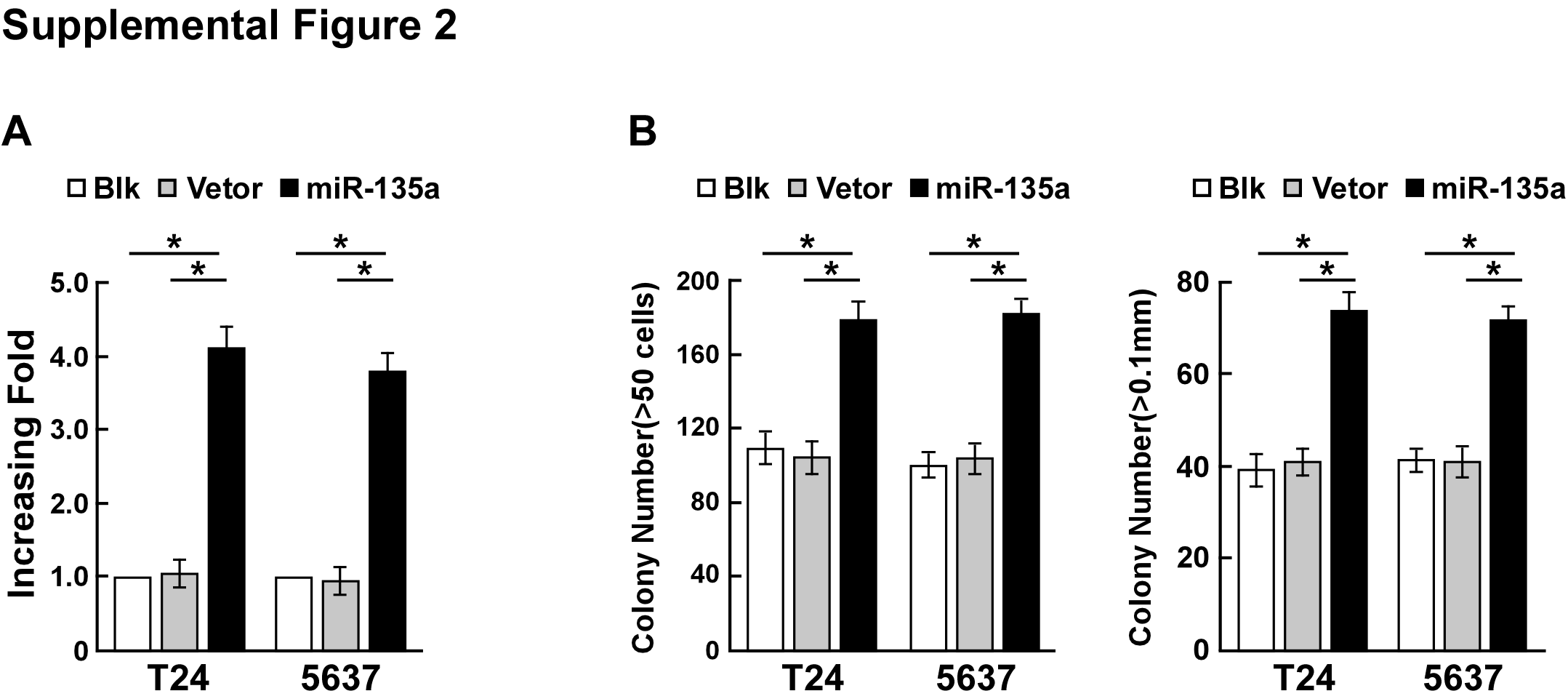

Supplement: Additional file 2: Figure S2. — MiR-135a induces proliferation of bladder cancer cells. A. Quantifications of crystal violet stained colonies formed by the indicated cells. B. Effects of miR-135a on the tumorigenicity of the indicated cells, as determined by anchorage-independent growth ability assays. All these experiments were done with T24 and 5637 cells stably overexpressing miR-135a, pMSCV-vector (presented as Vector) or non-transfected cell control (presented as Blk). Bars represent the mean ± SD of three independent experiments. *P <0.05. [file 12967_2015_438_MOESM2_ESM.tiff]

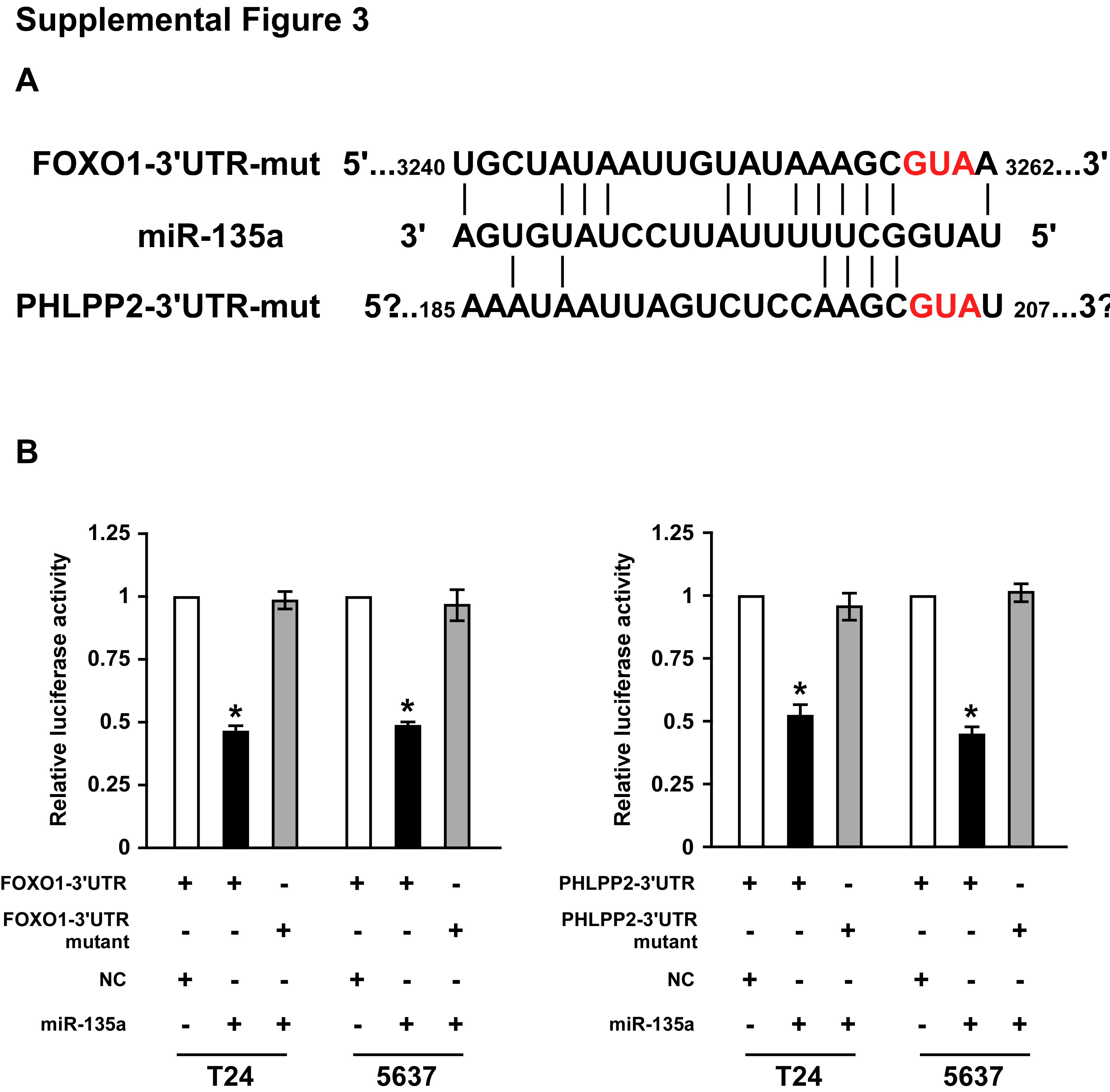

Supplement: Additional file 3: Figure S3. — FOXO1 and PHLPP2 are direct targets of miR-135a in bladder cancer cells. (A) Sequence of pGL3-FOXO1 or PHLPP2-3′UTR reporter with a mutant miR-135a binding site. (B) Luciferase assay of co-transfected with miR-135a or the control in the indicated cells. Experiments were repeated at least 3 times with similar results, and error bars represent ± SD, *P<0.05. [file 12967_2015_438_MOESM3_ESM.tiff]
